# Pacing of the first and only female finisher in the world's longest triathlon: The 2024 Triple Deca ultra triathlon

**DOI:** 10.1016/j.jesf.2026.200454

**Published:** 2026-01-19

**Authors:** Sasa Duric, Marilia Santos Andrade, Luciano Bernardes Leite, Pedro Forte, Pantelis T. Nikolaidis, Ivan Cuk, Katja Weiss, Thomas Rosemann, Beat Knechtle

**Affiliations:** a Liberal Arts Department, American University of the Middle East, Egaila, Kuwait; b Department of Physiology, Federal University of Sao Paulo, Brazil; c Department of Physical Education, Federal University of Viçosa, Viçosa, MG, Brazil; d Department of Sports, Higher Institute of Educational Sciences of the Douro, Penafiel, Portugal; e Department of Sports Sciences, Instituto Politécnico de Bragança, Bragança, Portugal; f Research Center for Active Living and Wellbeing (Livewell), Instituto Politécnico de Bragança, Bragança, Portugal; g School of Health and Caring Sciences, University of West Attica, Athens, Greece; h Faculty of Sport and Physical Education, University of Belgrade, Belgrade, Serbia; i InterSynergy Research Center, Belgrade, Serbia; j Institute of Primary Care, University of Zurich, Zurich, Switzerland; k Medbase St. Gallen Am Vadianplatz, St. Gallen, Switzerland

**Keywords:** Pacing strategy, Ultra-triathlon, Woman, Race time, Swimming, Running, Cycling

## Abstract

**Purpose:**

Pacing in triathlon has been analyzed for distances up to 60 long-distance triathlons in 60 days in men. However, no study has examined pacing in a female ultra-endurance triathlete in a multi-day triathlon exceeding 10 days. Thus, this case study analyzes the pacing of the first and only woman to complete 30 long-distance triathlons in 30 days.

**Methods:**

Lap times for swimming, cycling, and running, including transitions, were collected from race results. The athlete tracked each discipline daily using a Fenix 7 Sapphire Solar, recording average and maximum heart rates and energy expenditure. The coefficient of variation and second-order polynomial regression were calculated for average pace, split, and total times. Repeated measures ANOVA tested interactions in pace performance across 10-day phases and intra-discipline daily pacing variations. Multivariate regression examined physiological parameters’ impact on pacing.

**Results:**

The female triathlete maintained a relatively even pacing strategy throughout the race, with a decrease in cycling speed and an increase in running speed. Cycling showed the strongest and significant correlation with total race time (r = 0.810; p < 0.001), while running (r = 0.347; p = 0.119) and swimming (r = −0.312; p = 0.165) displayed non-significant associations. The pace varied within the disciplines, with cycling becoming slower and running faster in the last quarter of the race. Energy expenditure, maximum and average heart rate were significant predictors for cycling (R^2^ = 0.538; p < 0.001), while only average heart rate was the best predictor for running performance (R^2^ = 0.450; p < 0.001).

**Conclusions:**

Tactical considerations most likely influenced pacing, particularly in cycling and running. Future research should further explore pacing strategies in ultra-endurance events.

## 1 Introduction

Ultra-triathlons are long-distance triathlon races covering x-times the IRONMAN® triathlon of 3.8 km swimming, 180 km cycling and 42.195 km running. Therefore, a Double Iron ultra-triathlon covers two times, a Triple Iron ultra-triathlon three times, a Quintuple Iron ultra-triathlon five times, and a Deca Iron ultra-triathlon ten times these distances.^1^, ^2^, ^3^ These races can be held as a continuous version such as the classical IRONMAN® triathlon, but also as a per day version where daily an IRONMAN® triathlon has to be completed.^1^, ^2^, ^3^

Generally, men are faster than women in ultra-endurance events such as IRONMAN® triathlons^4^ and ultra-triathlons.^5^ However, in some instances at races at world-class level, the best women can swim and cycle faster than the best men.^6^ The better performance in male ultra-endurance athletes is mainly due to the lower participation of female ultra-endurance athletes.^7^

Although women have a greater proportional area of type I fibers, are more able to use fatty acids and preserve carbohydrates during prolonged exercise with a greater substrate efficiency, use a more even pacing strategy and have a greater fatigue resistance, other factors such as lower O_2_ carrying capacity, greater body fat percentage counterbalance these potential advantages, making women outperforming men a rare exception.^8^^,^^9^

To date, pacing in ultra-triathlon races has been investigated in the continuous version of Double Iron, Triple Iron, Quintuple Iron and Deca Iron ultra-triathlons.^1^ In the per day version, one study has analyzed the pacing of cycling and running in male triathletes competing in a Quintuple Iron, Deca Iron and Double Deca Iron ultra-triathlon.^2^ Regarding the longest race distance, the Triple Iron ultra-triathlon, one study has compared Deca Iron and Triple Deca Iron ultra-triathletes regarding the changes in split and overall race times over the days.^3^

Over longer distances than the Triple Deca Iron ultra-triathlon, only men could finish more than 30 IRONMAN®-distance triathlons. One man was able to finish 60 IRONMAN®-distance triathlons within 60 days^10^ and another man even completed 100 IRONMAN®-distance triathlons in 100 days.^11^^,^^12^ Recently, another man completed 120 IRONMAN®-distance triathlons in 120 days.^13^

Comparing to men, women form a relatively small group in ultra-triathlon races,^3^^,^^5^^,^^6^ despite the fact they might perform better than men.^9^ In terms of pacing in multi-day triathlons such as the Quintuple or Deca Iron ultra-triathlon, there is a case report of a female athlete who set a new world record in the Quintuple and Deca Iron ultra-triathlon. This athlete showed an even cycling and running pacing in both records with no variations within- or between days.^14^

In 2024, a woman finished a Triple Deca Iron ultra-triathlon (30 IRONMAN®-distance triathlons in 30 days) as a solo athlete, with all men dropping out of the race.^15^ To better understand the reasons why this woman was able to accomplish the record, it is important to understand the racing strategy. Some studies focus on biomechanical and pacing variables,^16^ physiological demands,^17^ and psychological factors.^18^ However, it is important to highlight that in addition to the different factors that contribute to performance, pacing is the determinant variable that describes the performance (as fast as possible) and the strategy to accomplish the speed record.

The aim of this case study was, therefore, to analyze pacing in all three split disciplines, to investigate which discipline is the best predictor of overall race outcome, and to investigate the relationships between physiological parameters such as energy expenditure, average heart rate and maximum heart rate in swimming, cycling and running. We hypothesized that this female athlete would also adopt an even pacing strategy, and we further explored whether female pacing patterns differ from known male strategies.

## 2 Method

### 2.1 Athlete with experience and pre-race preparation

The athlete is a female ultra-endurance athlete (born 1971, 164 cm, 67 kg) from Austria. In ultra-cycling, she finished the Race Across AMerica (RAAM) in 2017 in 12:04:35 d:h:min in second place in the women's race and in 2019 in 12:05:41 d:h:min in third place.^19^ In 2019, she also set a world record in the Race Across Australia (3966 km) in 9:12:33 d:h:min and two world records with 13,333 km in 30 days and 3953.42 km in one week. In the ultra-triathlon, she set a world record in 2016 in the Double Deca one per day in 279:37:48 h:min:s, in 2018 in the Quintuple one per day in 84:44:29 h:min:s, in 2022 in the Deca one per day in 137:23:43 h:min:s, and in 2023 in the Double Deca continuous race in 554:56:32 h:min:s.^20^

In the pre-race preparation (start January 2024), she invested ∼21 hours per week in training, mainly in cycling. In winter she invested ∼15 hours per week, in summer ∼25–30 hours per week. In winter, she mainly did indoor cycling on a Tacx® NEO Bike Smart-Trainer. She swam an average of ∼7.9 km per week and ∼34.3 km per month. For cycling, she invested ∼16 hours per week and ∼70 hours per month. For running, her weekly running kilometers are ∼45.1 km and her monthly kilometers are ∼195.5 km. The training was mainly divided into an early morning session before work and a second session in the evening after work. Monthly cycling kilometers varied between 1069 km (August) and 2073 (June) while monthly running kilometers varied between 81 km (April) and 217 km (June).

### 2.2 The race

The race took place near Desenzano del Garda in Northern Italy, with the first day (Day 1) on September 5 and the last day (Day 30) on October 4, 2024 (21). The athlete had to complete a daily IRONMAN®-distance triathlon covering 3.8 km swimming, 180 km cycling and 42.195 km running. The race headquarters was located at the “Le Ninfee” aquatic park, which features a 25-m lane pool (not heated) and a 1.06 km flat running track on dirt, grass, sand, and asphalt. The 7 km bike loop was located at a separate location 11.5 km from “Le Ninfee” by bike, and one has to complete this out-and-back and 23 seven-kilometer loops to get to the required 180 km bike ride. The athletes had to overcome an average accumulation of ∼1000 m uphill and ⁓1000 m downhill over the 180 km. When running, the athletes had to complete 40 laps. For swimming, she wore a Sailfish® wetsuit; for cycling, she used a Ridley® Noah SL with Pancho® wheels with 8 cm wide rims. For cycling, she wore cycling pants from Cocoon® and a cushion from Tempur® for her bicycle saddle. For running, she started with HOKA® Speed Goat on day 1 but then switched to TOPO® ATMOS for the remaining 29 days.

At the start, the environmental conditions were quite good, with a water temperature of ∼22 °C and sunshine. As the days progressed, the water temperature dropped steadily to ∼19 °C in the middle of the race and to ∼17–18 °C in the last week. The air temperature varied between 20 and 25 °C, depending upon rainy or sunny days. A total of 7 athletes (6 men and 1 woman) started the race on September 5, 2024. The first man dropped out on day 1, the second man on day 3, the third man on day 4, the fourth man on day 5, the fifth man on day 10, and the last man on day 16. From day 17 to day 30, the female athlete was alone in the race.

### 2.3 Data set and data preparation

The race data with split and lap times for swimming, cycling, and running were obtained from the official race website of the ‘Ultra Triathlon Italy’.^21^ The lap times for swimming were recorded manually, and the final swim time was provided. Cycling and running lap times were recorded electronically using a chip system (www.raceresult.com/). The athlete monitored each split discipline (*i.e.* swimming, cycling, and running) of each day using the Fenix® 7 Sapphire Solar. The recordings of average heart rate, maximum heart rate and energy expenditure for the three split disciplines were extracted for each phase. For further statistical analysis, we used the data of each discipline from each day, so that we formed 30 data sets for each discipline, which allowed us to perform some meaningful statistical analyses.

### 2.4 Statistical analysis

The normality of the distribution of the dependent variables was tested using the Kolmogorov-Smirnov test. The descriptive data are given as mean, standard deviation (SD) and range (min-max). In addition, the coefficient of variation (CV) and the coefficient of determination of the second-order polynomial regression (R^2^) were calculated for the average pace values, split times, and total times of the race. Repeated-measures ANOVA models were conducted with Race Phase (Days 1–10, 11–20, 21–30) as the within-subject factor. Dependent variables included split times (min) for swimming, cycling, running, transitions (T1, T2), and total race time. For within-discipline pacing analysis, a secondary within-subject factor, Discipline Segment (Swim = 1 km, 2 km, 3 km, 3.8 km; Bike/Run = Quarter 1–4), was included. Greenhouse–Geisser corrections were applied when sphericity was violated, and effect sizes were reported as partial eta squared – η^2^. Partial eta squared was interpreted as 0.01, 0.06 and 0.14 indicating small, medium and large effects for each factor, respectively.^22^^,^^23^ Furthermore, a backward stepwise multivariate regression analysis was performed for physiological parameters such as energy expenditure, average heart rate, and maximum heart rate to test their impact on swimming, cycling, and running performance. In addition, the same analysis was performed for split times in swimming, cycling, and running to predict overall performance. All correlation coefficients were interpreted as small, r = 0.10–0.29; moderate, r = 0.30–0.49; and large, r = 0.50–1.0 (**12**). The significance level was set at p < 0.05. Statistical Software for the Social Sciences was used for all statistical procedures (SPSS v25, Chicago, Illinois, USA).

## 3 Results

**Fig. 1:**
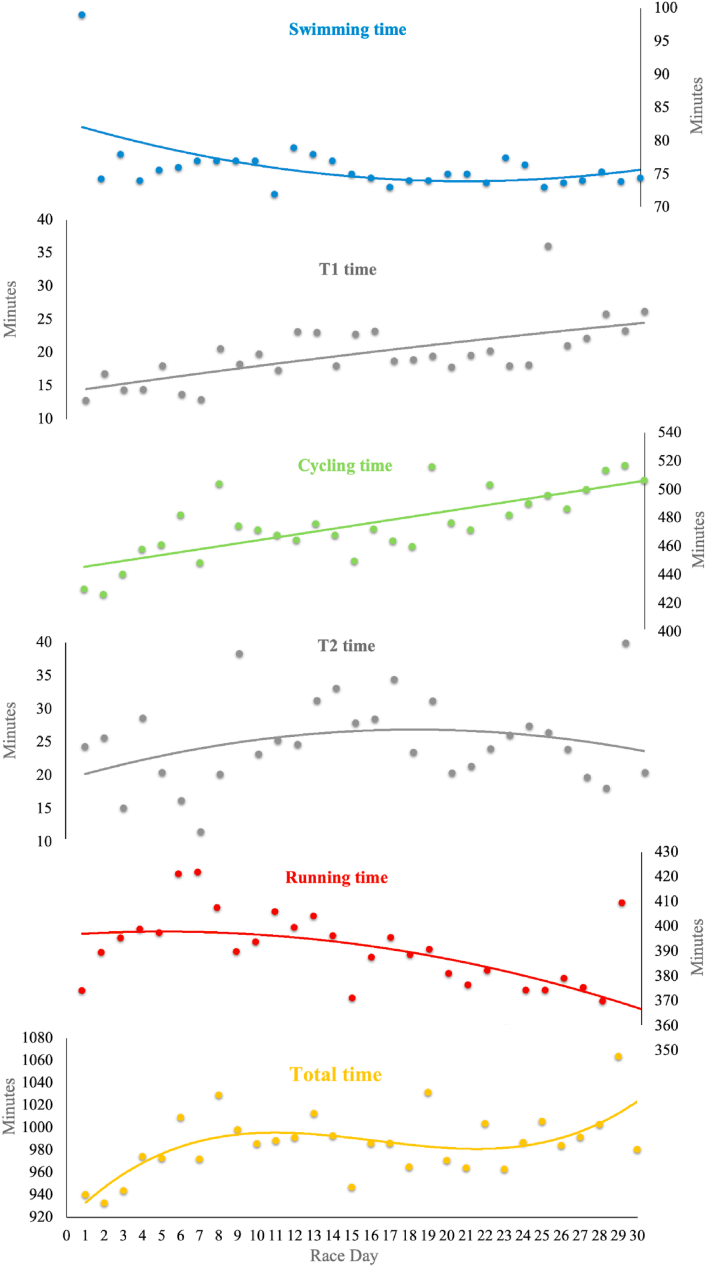
Pacing patterns across 30 consecutive race days for swimming, transition time - T1, cycling, transition time - T2, running, and total race time in 30 Triple Deca Ultra Triathlons.

The Kolmogorov-Smirnov test showed that the data were normally distributed. The multivariate regression analysis with total time as the dependent variable and swimming, cycling, and running performance as independent variables showed statistical significance (p < 0.001) with an adjusted R^2^ of 0.915, as expected. The highest and significant correlation was observed between cycling performance and overall race time (r = 0.810, p < 0.001), whereas running (r = 0.347, p = 0.119) and swimming performance (r = −0.312, p = 0.165) displayed small-to-moderate, non-significant associations. Second-order nonlinear regressions showed the best fit for cycling split time, but little or no fit models for swimming, running and total race time. The coefficients of variation (CV) were higher for swimming, followed by cycling and running (Table 1). For overall times, the penultimate day was the slowest and the slowest day for cycling performance and T2 (Fig. 1). A third-order non-linear polynomial regression was the best fit for overall performance (R^2^ = 0.378).

**Table 1:** Split, overall and transition times (minutes), coefficient of variance (CV) and goodness of fit of second-order polynomial regression (R^2^) for the 30 Triple Deca triathlon distances over 30 consecutive days.

Times (min)	Mean (SD)	Min-Max	CV (%)	R^2^
Swimming split	76.1 (4.7)	72.0–99.0	6.1	0.27
T1	19.8 (4.7)	12.8–36.0	23.5	0.43
Cycling split	476.0 (24.3)	426.2–517.0	5.1	0.57
T2	25.0 (6.5)	11.6–40.0	25.9	0.08
Running	388.8 (16.6)	352.9–422.0	4.3	0.35
Total	985.7 (28.2)	932.4–1063.8	2.9	0.18

T1 = first transition; T2 = second transition.

**Fig. 2:**
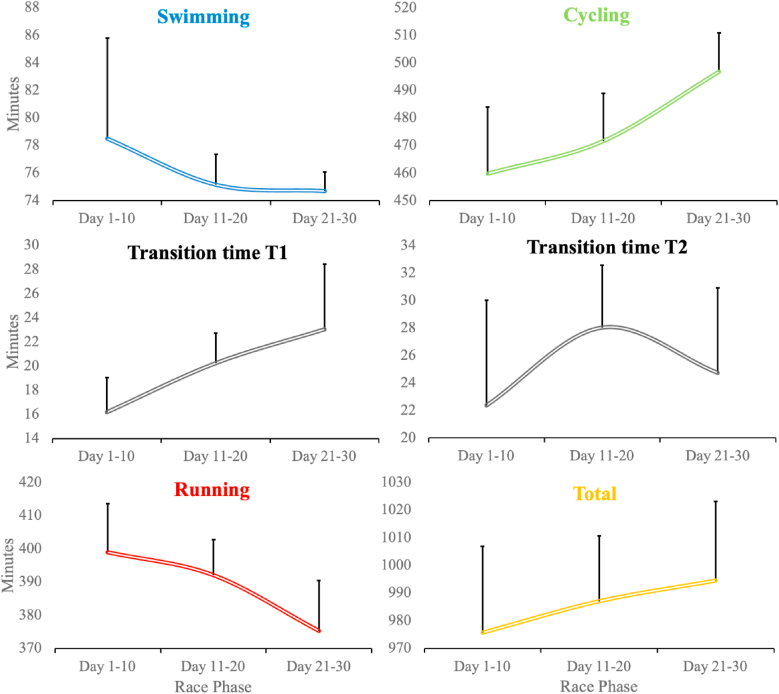
Ten-day mean times for swimming, cycling, running, transition times and total time for 30 Triple Deca ultra-triathlon distances. *p < 0.05, **p < 0.01 statistical significance for time effect (race phase).

The repeated measures ANOVA indicated significant time effects across race phases for cycling (F = 24.1; η_p_^2^ = 0.858; p < 0.001), running (F = 6.2; η_p_^2^ = 0.608; p = 0.023), and T1 transition times (F = 14.3; η_p_^2^ = 0.781; p < 0.002). In contrast, no significant time effects were observed for swimming, T2, or overall pacing performance (Fig. 2).

As shown in Fig. 2, the average cycling pace in the final phase (days 21–30) was significantly lower (lower velocity) than the average cycling pace in the opening phase (days 1–10) of the race (Fig. 2). Significant differences were also observed in running, where the pace increased (higher velocity) over the course of the race. Thus, the average running pace in the final phase (days 21–30) was significantly higher than the average pace in the middle (days 11–20) and in the opening phase (days 1–10) of the race.

**Fig. 3:**
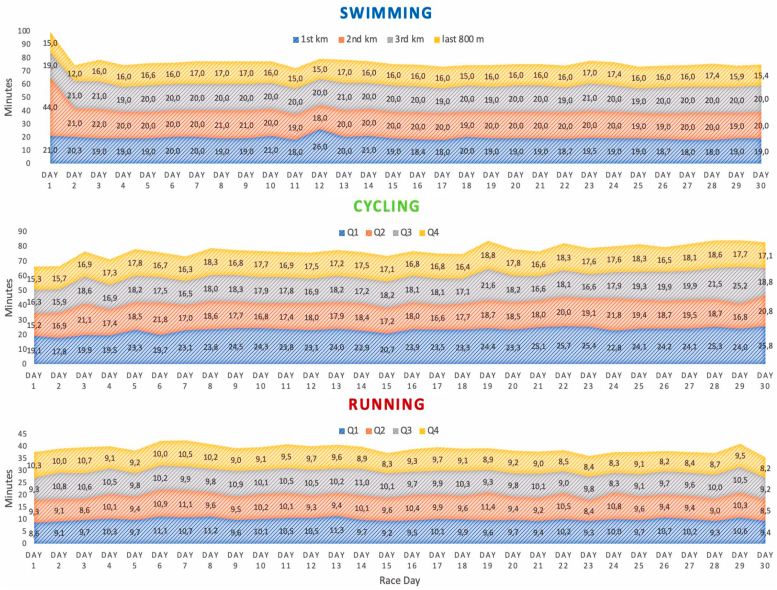
Average daily pace expressed by the average split times in swimming (1st km, 2nd km, 3rd km, last 800 m split times), cycling, and running (mean quarterly split times Q1, Q2, Q3, Q4).

In addition to the overall split times, the daily split times were also observed and analyzed (Fig. 3).

**Fig. 4:**
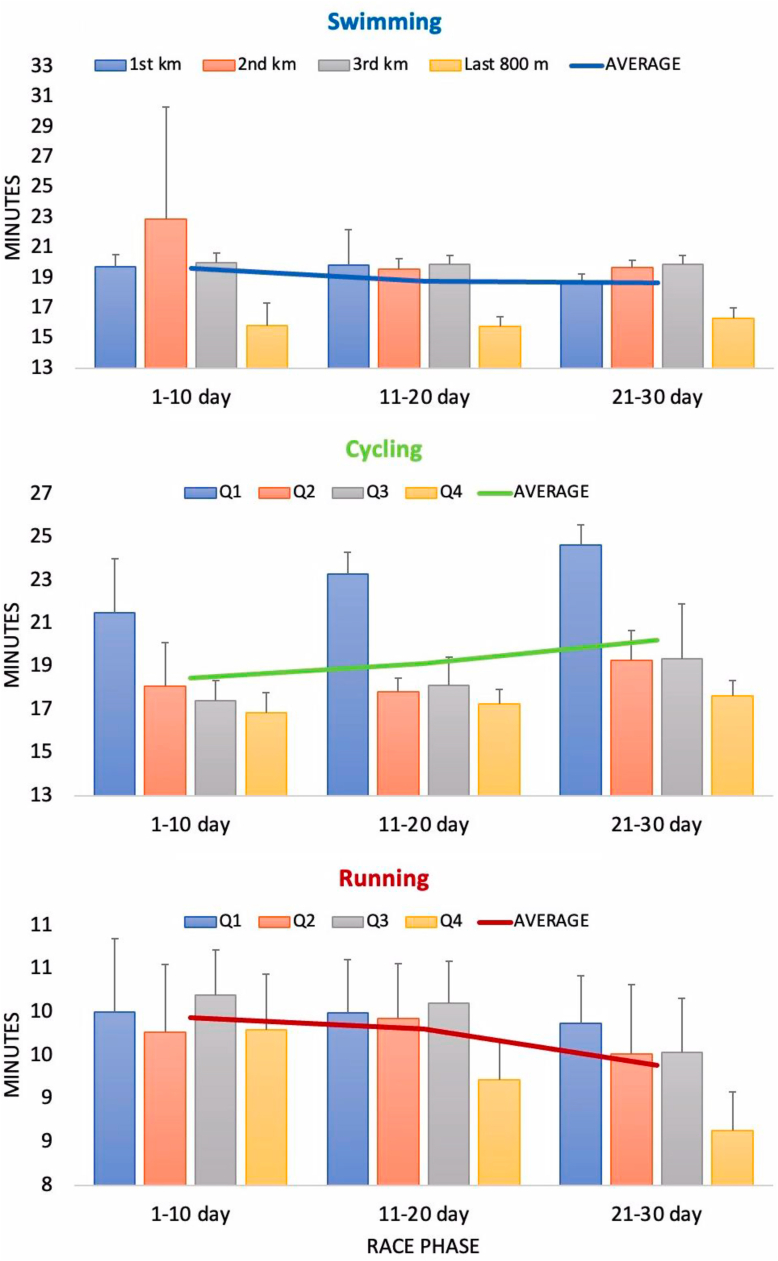
Ten-day mean daily split times for swimming (1st km, 2nd km, 3rd km and last 800 m), cycling (Q1, Q2, Q3 and Q4) and running (Q1, Q2, Q3 and Q4) at 30 Triple Deca ultra-triathlon distances.

In addition, swimming times were analyzed for the 1st, 2nd^,^ and 3rd kilometers and for the last 800 m, while cycling and running times were analyzed for 4 quarters (Q1, Q2, Q3 and Q4) of each day/race, averaged over three 10-day race phases – opening, middle and final (Fig. 4).

The repeated measures ANOVA (with the factors *race phase* and *discipline phase*) showed that in swimming the *discipline phase* factor was significant, while the *race phase* was not significant and there were no significant interactions between the factors. A further post-hoc analysis showed that there were differences between the final 800 m split time and the other split times - 1 km, 2 km and 3 km (overall p < 0.001), which was to be expected as 800 m is a shorter distance than 1 km. There were no significant differences between the other average split times.

For cycling, the two factors *race phase* and *discipline phase* and their interaction were significant (p = 0.001, F = 20.909, η_p_^2^ = 0.839; p < 0.001, F = 165.791, η_p_^2^ = 0.986 and p = 0.034, F = 7.712, η_p_^2^ = 0.920, respectively). A more detailed post-hoc analysis showed that the final race phase was significantly slower than the opening phase (p = 0.010) and the middle phase (p = 0.006). As for the *discipline phase*, Q1 of the race was significantly slower than the rest (p < 0.001), and Q2 was significantly slower than Q4 (p = 0.014).

In running, the two factors *race phase* and *discipline phase* were significant (p = 0.036, F = 5.192, η_p_^2^ = 0.565 and p < 0.001, F = 26.169, η_p_^2^ = 0.918, respectively), but not their interaction (p = 0.165). Surprisingly, the post-hoc analysis showed that the pace was significantly faster in the final phase of the race (21–30 day) than in the opening phase (1–10 days; p = 0.039) and the middle phase (11–20 days; p = 0.048). Similarly, on average, the last part of the running phase (Q4) was attributed a significantly faster pace than the earlier parts - Q1 and Q3 (p = 0.001 and p < 0.001, respectively).

Table 2 contains three multivariate regression models, one for swimming, one for cycling and one for overall running performance as the dependent variable and physiological parameters such as average heart rate, maximum heart rate and energy expenditure. The first regression model used swimming as the dependent variable and showed no statistical significance (p = 0.427) with an adjusted R^2^ of −0.004. The highest correlation was found with energy expenditure with r = 0.136. The second regression model with cycling as the dependent variable showed statistical significance (p < 0.001) with an adjusted R^2^ of 0.538. The highest positive correlation was again observed between energy expenditure and overall performance (with r = 0.413). The standardized regression coefficient (B = 0.539) indicates a strong positive association between average heart rate and cycling performance. Practically, this means that a one standard deviation decrease in average heart rate was associated with approximately a 0.54 standard deviation improvement in cycling time, suggesting that maintaining a lower physiological strain across race days contributed meaningfully to faster cycling performance. The third regression model with running as the dependent variable also showed statistical significance (p < 0.001) with an adjusted R^2^ of 0.430. However, when using the stepwise backward approach, the analysis showed that the adjusted R^2^ for the model included only maximum and average heart rate; finally, only average heart rate had the adjusted R^2^ of 0.450. Thus, the highest correlation was observed between the average heart rate and the overall running performance, with r = −0.685.

**Table 2:** Multivariate regression analysis to assess the influence of physiological variables on swimming, cycling, and running split times in 30 Triple Deca ultra-triathlon distances.

Model (Dependent variable)	Independent variables	Standardized B	p-value
Swimming	Energy expenditure (kcal)	0.587	0.108
	Average heart rate (bpm)	−0.595	0.259
	Maximum heart rate (bpm)	0.068	0.878
Cycling	Energy expenditure (kcal)	1.589	<0.001
	Average heart rate (bpm)	−1.722	<0.001
	Maximum heart rate (bpm)	0.539	0.014
Running	Energy expenditure (kcal)	0.045	0.879
	Average heart rate (bpm)	−0.805	<0.001
	Maximum heart rate (bpm)	0.145	0.601

## 4 Discussion

This case study examined the pacing of the first and only woman to finish a Triple Deca Iron ultra-triathlon (30 IRONMAN®-distance triathlons in 30 days) in which all men dropped out of the race. We hypothesized that this athlete would have an even pacing strategy. The main findings were: (i) the athlete predominantly adopted an even pacing strategy, with cycling showing the strongest correlation with total race time, followed by running and swimming performance; (ii) pacing varied across the 30 days in both cycling and running; (iii) differences in pacing were observed between discipline phases and race phases within the split disciplines; and (iv) correlations between physiological variables and split performances differed across disciplines.

### 4.1 Cycling as the key discipline of the race

The first important finding was that cycling had a higher correlation with the overall race time than running and swimming. Although small-to-moderate correlations were observed for running and swimming, these did not reach statistical significance (p > 0.05). Consequently, their apparent relationships with total performance should be viewed as descriptive trends rather than inferential findings. The three split disciplines seemed to have a different predictive influence on overall triathlon race time depending upon the length of the triathlon race (24). In general, cycling seems to be the most predictive split discipline in an IRONMAN® triathlon,^24^ while swimming has the least impact on the IRONMAN® race performance.^25^ Although cycling naturally accounts for the largest share of total race time, which can partly explain its strong relationship with total performance, our correlation analysis was conducted using absolute split times (not relative time percentages). When expressed as standardized paces (min·km^−1^), cycling continued to show the strongest association with total time, indicating that beyond its proportional duration, variability in cycling performance most strongly influenced the athlete's overall outcome. However, in some studies, running had a similar^25^ or even higher impact^26^ than cycling. These differences are likely due to different samples (e.g. recreational or professional athletes) used for the analysis.

### 4.2 Differences in cycling and running pace over the 30 days

A second important finding was that the athlete adopted a different pacing in cycling and running. While the average cycling pace in the final phase (days 21–30) was significantly lower than the average cycling pace in the opening phase (days 1–10) of the race, the average running pace in the final phase (days 21–30) was significantly higher than the average pace in the middle (days 11–20) and in the opening phase (days 1–10) of the race. The athlete gradually reduced her cycling speed as the race progressed but increased her running speed toward the final stages. A potential explanation for this could be the change in environmental conditions since the start of the race in early September and the end of the race in early October, where outside temperatures may have dropped during these days.^27^

However, tactical considerations might also have led to this change in pace. The athlete has set several records in ultra-cycling and may have reduced her pace in cycling to save energy for the run.^25^ It has been reported that running performance was impaired when running was performed after cycling.^28^ It has also been shown that mechanical efficiency of running is decreased and anaerobic energy expenditure is increased when a 40-km bout of cycling is performed immediately before running 5 km.^29^ Motivation could also have played an important role since the woman was aware to be the first and only woman in the world to be able to finish such a race.^30^

### 4.3 Differences between the discipline phase and the race phase for the split disciplines

A third important finding was that there were pacing differences between the discipline and race phases. While only the discipline phase factor was significant in swimming, the discipline and race phases were significant in cycling and running. Furthermore, in cycling, the final race phase was significantly slower than the opening phase and the middle phase; whereas in running, the pace was significantly faster in the final phase than in the opening and middle phases of the race. In other words, this analytical approach also showed a change in pacing over the race in cycling (decrease in speed) and running (increase in speed). However, this strategy might be typical for ultra-triathletes competing in this race format. A male athlete who completed 40 IRONMAN®-distance triathlons in 40 days also changed cycling and running speeds across days, with cycling times becoming slower across days. In contrast, running times became faster by day 20 and then slower by day 40.^27^

### 4.4 Different correlations with physiological variables and split performances

A key final finding was that the physiological variables measured during the event (e.g., heart rate and energy expenditure) showed distinct associations with split performances. Energy expenditure was correlated with swimming and cycling performance, but not with running performance, where average heart rate and overall running performance were correlated. These differences could be a reflection of the length of an endurance event. In a male triathlete who completed 20, 40 and 60 IRONMAN®-distance triathlons, differences in heart rate were also found across the different events and disciplines.^10^

### 4.5 A woman defeats all men

In this race, our female athlete was the only one to finish the race, and no man could finish. An explanation for this could be her vast previous experience of finishing ultra-cycling races like the RAAM or her world records for the highest monthly and weekly mileage. But also, her world records for shorter ultra-triathlon distances. Previous experience has been shown to be an important predictor in triathlon.^31^^,^^32^ A study investigating ultra-triathletes competing in Double, Triple, Quintuple and Deca Iron ultra-triathlon showed that previous experience was important for performance in longer ultra-triathlon races (i.e. Quintuple and Deca Iron ultra-triathlon), while personal best times were important in shorter ultra-triathlon races such as the Double and the Triple Iron ultra-triathlon, but the number of previously finished races was not.^33^ For Deca Iron ultra-triathletes, personal best time in a Triple Iron ultra-triathlon was also an important predictor.^34^ For shorter triathlon distances such as the IRONMAN® triathlon distance, triathletes with previous experience in an IRONMAN® race achieved faster overall race times.^35^ For IRONMAN® triathlon performance in particular, previous best performance in an Olympic-distance triathlon was an important predictor for both female and male triathletes.^31^^,^^36^^,^^37^ Previous experience is not only important for triathletes and ultra-triathletes, but also for ultra-marathoners. Among the successful finishers of the TransEurope FootRace 2009, pre-race records in specific ultra-marathons events such as 6-h, 50-km and 100-km races showed a high correlation with performance in the TransEurope FootRace.^38^ In addition to previous experience, some differences in physiological characteristics may also favor women in long-duration competitions, such as a higher percentage of oxidative fibers and greater utilization of fats as an energy substrate.^39^^,^^40^

Moreover, it seemed that the participant's sex was not a limiting factor of performance considering the evidence of small sex differences in ultra-endurance performance (e.g., 12 % in IRONMAN® World Championships 2014–2023,^4^ 8.5 % in IRONMAN® 70.3 2004–2020,^41^ and 9.6 % in 12h and 21.9 % in 24h ultra-cycling, respectively.^42^ Indeed, elite women are able to beat elite men in ultra-endurance triathlon races at world-class level in split disciplines. This might be due to the fact that a highly selected and committed female cohort competes in race at world-class level where they achieve a high completion race.^6^

It is important to note that this case study has some important aspects to highlight. This study's strengths include the detailed analysis of pacing strategies in a female ultra-endurance triathlete completing the Triple Deca Ultra-Triathlon, the use of objective physiological data from heart rate and energy expenditure monitoring, and the statistical modeling that identified cycling as the key determinant of overall performance. Additionally, the study offers valuable insights into sex differences in ultra-endurance racing and the pacing adaptations over the 30-day event.

Finally, this study presents some limitations. The case study design restricts the generalizability of the findings to other female ultra-triathletes. Additionally, environmental factors such as temperature fluctuations were not controlled, and the lack of data on nutritional intake, hydration, sleep, or psychological factors limits a comprehensive understanding of performance determinants. Heart rate and energy expenditure were measured using a Fenix 7 smartwatch. Heart rate data of a broad variety of smartwatches used in triathlon training has recently been shown to be invalid for a substantial share of athletes if the built-in optical measurement methodology is used.^43^ Similarly, energy expenditure estimates of the Fenix 7 smartwatch are highly approximate.^44^ Including environmental conditions such as temperatures, humidity, and barometric pressure would be helpful in order to find a potential influence of environmental factors.^45^ Therefore, future research should include larger samples of ultra-endurance athletes and explore the interaction between physiological, environmental, nutritional, and psychological factors in pacing strategies, and investigate the long-term adaptations resulting from multi-day ultra-triathlon participation. Qualitative interviews or self-reflection diaries could be incorporated to complement pacing data. Such a mixed-methods approach would allow exploration of psychological and motivational processes underlying pacing adjustments, particularly after the athlete became the sole remaining competitor after Day 16. In addition, this case study is based on a single participant; therefore, inferential statistics such as ANOVA and regression are interpreted descriptively. Results should be viewed as representative of one athlete's pacing dynamics and not extrapolated to the broader triathlon population. Future studies using improved devices with higher accuracy in measuring physiological parameters would further enhance the validity of similar investigations.^46^

## 5 Conclusion

In summary, the observed female triathlete maintained an even pacing strategy throughout the race. Cycling pace decreased while running pace increased, likely due to tactical considerations, whereas swimming pace remained stable over 30 days. Cycling showed the strongest correlation with total race time, followed by running and swimming. Pacing varied across discipline phases, with a slower cycling pace and a faster running pace in the final quarter. Additionally, energy expenditure and heart rate were significant predictors of cycling performance, while heart rate predicted running performance.

## Declaration of competing interest

The authors declare that they have no known competing financial interests or personal relationships that could have appeared to influence the work reported in this paper.
